# Supplementary material for: Physiotherapy Improves Dogs’ Quality of Life Measured with the Milan Pet Quality of Life Scale: Is Pain Involved?
Source: Vet Sci. 2022 Jul 2;9(7):335. doi: 10.3390/vetsci9070335 (PMC9324735; doi:10.3390/vetsci9070335)
Supplement: Supplementary file 1 [file vetsci-09-00335-s001.zip › vetsci-1776693-supplementary.pdf]

Table S1. Demographic data of the dogs

Name	Age (years)	Sex	Breed	Condition	Treatment	Duration (weeks)
**Wilson**	1	Intact male	Australian shepherd	Orthopaedic	Hydrotherapy	4
**Ettore**	4	Intact male	French bulldog	Neurological	Hydrotherapy & Instrumental	4
**Yoda**	8	Neutered female	Pug	Neurological	Hydrotherapy	4
**Osvaldo**	8	Intact male	Boxer	Neurological	Hydrotherapy & Instrumental	4
**Lara**	10	Neutered female	Italian bloodhound	Orthopaedic	Hydrotherapy	4
**Ryan**	1	Intact male	Border collie	Orthopaedic	Hydrotherapy & Instrumental	6
**Tommy**	14	Intact male	Poodle	Orthopaedic	Instrumental	4
**Margot**	4	Neutered female	Australian shepherd	Orthopaedic	Hydrotherapy & Instrumental	4
**Serse**	4	Neutered male	Cane corso	Degenerative	Hydrotherapy & Instrumental	4
**Kira**	5	Neutered female	Mixed breed	Orthopaedic	Hydrotherapy & Instrumental	4
**Ettore**	4	Intact male	Shar-pei	Neurological	Hydrotherapy	5
**Trevor**	5	Neutered male	Mixed breed	Orthopaedic	Hydrotherapy	3
**Erik**	2	Intact male	Cane corso	Orthopaedic	Hydrotherapy	6
**Idylle**	7	Neutered female	Belgian shepherd terveuren	Orthopaedic	Hydrotherapy & Instrumental	4
**Lucy**	5	Neutered female	Mixed bred	Orthopaedic	Hydrotherapy & Instrumental	4
**Picci**	4	Neutered female	American bulldog	Orthopaedic	Hydrotherapy & Instrumental	4
**Arya**	5	Intact female	Spanish greyhound	Orthopaedic	Hydrotherapy & Instrumental	4
**Locki**	4	Intact male	Siberian husky	Orthopaedic	Hydrotherapy	4
**Pedro**	11	Neutered male	Labrador retriever	Degenerative	Hydrotherapy & Instrumental	7
**Cody**	1	Intact male	Poodle	Degenerative	Hydrotherapy	6

Table S2. Medical checklist. For each time (T_0 and T_1) median value and range (in brackets) are reported.

Variable	Definition	T_0	T_1
**BCS**	Body Condition Score<br>1=underweight<br>2=normal weight<br>3=overweight	2 (2-3)	2 (2-3)
**Appetite**	1=improved<br>2=same as before<br>3=worsened	3 (1-3)	3 (1-3)
**Playfulness**	1=improved<br>2=same as before<br>3=worsened	1 (1-3)	2 (2-3)
**Interaction with owner**	1=improved<br>2=same as before<br>3=worsened	1 (1-3)	2 (1-2)
**Walking**	1=improved<br>2=same as before<br>3=worsened	1 (1-3)	2 (1-2)
**Lameness**	1=none<br>2=mild<br>3=moderate<br>4=severe	2 (1-4)	1 (1-3)
**Pain**	1=none<br>2=mild<br>3=moderate<br>4=severe	2 (1-4)	1 (1-3)
**Movement**	1=improved<br>2=same as before<br>3=worsened	1 (1-3)	1 (1-2)
**Criticality**	1=mild<br>2=moderate<br>3=severe	2 (1-3)	1 (1-2)

Table S3. MQPL norms for each time of assessment (T_0 vs T_1).

Domain	T_0				T_1			
	**Mdn (Min-Max)**	**SE**	**Skeweness**	**Kurtosis**	**Mdn (Min-Max)**	**SE**	**Skeweness**	**Kurtosis**
**Physical**	61 (50-72)	1.24	0.04	-0.55	62 (56-72)	0.83	0.52	0.06
**Psychological**	120 (95-130)	1.34	-0.86	-0.03	128 (116-138)	0.96	0.02	-0.93

**Social**	10 (8-13)	0.42	0.26	-1.55	11 (8-13)	0.43	-0.11	-1.78
**Environmental**	38 (24-44)	1.19	-0.61	-0.50	38 (27-45)	0.96	-0.39	-0.40

Table S4. Variables selections for the regression models.

Predictor	QOL physical	QOL psychological	QOL social	QOL environmental
Age[a]	r = -0.21, *p* = 0.182	**r = 0.78, *p* = 0.046**	r = 0.21, *p* = 0.192	r = 0.25, *p* = 0.115
Sex[b]	χ2 (df=3)=7.66, *p*=0.053	χ2 (df=3)=3.64, *p*=0.303	χ2 (df=3)=7.48, *p*=0.058	χ2 (df=3)=4.45, *p*=0.217
Medical problem[b]	χ2 (df=2)=4.60, *p*=0.100	χ2 (df=2)=1.00, *p*=0.606	**χ2 (df=2)=8.51, *p*=0.014**	**χ2 (df=2)=6.88, *p*=0.032**
Lameness[b]	χ2 (df=3)=5.95, *p*=0.114	χ2 (df=3)=1.24, *p*=0.744	**χ2 (df=3)=13.64, *p*=0.003**	χ2 (df=3)=0.19, *p*=0.978
Pain[b]	**χ2 (df=3)=7.97, *p*=0.046**	χ2 (df=3)=7.03, *p*=0.071	χ2 (df=3)=4.15, *p*=0.246	χ2 (df=3)=3.49, *p*=0.322
Movement[b]	χ2 (df=2)=3.54, *p*=0.170	χ2 (df=2)=1.92, *p*=0.382	χ2 (df=2)=0.77, *p*=0.680	χ2 (df=2)=4.74, *p*=0.093
Criticality[b]	**χ2 (df=2)=7.47, *p*=0.024**	χ2 (df=2)=5.45, *p*=0.066	χ2 (df=2)=1.71, *p*=0.426	χ2 (df=2)=0.49, *p*=0.782
Treatment[b]	χ2 (df=2)=1.35, *p*=0.509	χ2 (df=2)=0.77, *p*=0.680	χ2 (df=2)=3.20, *p*=0.202	χ2 (df=2)=0.79, *p*=0.674
Time[b]	χ2 (df=1)=3.42, *p*=0.064	**χ2 (df=1)=10.56, *p*=0.001**	χ2 (df=1)=0.65, *p*=0.420	χ2 (df=1)=0.11, *p*=0.744

Note: bold = $p < 0.05$, [a] = Spearman correlation, b = Kruskal-Wallis rank sum test.
